# Editorial: Translational Virology in Pregnancy

**DOI:** 10.3389/fviro.2022.908471

**Published:** 2022-05-23

**Authors:** Kristina M. Adams Waldorf, Vikki M. Abrahams

**Affiliations:** 1 Department of Obstetrics and Gynecology, University of Washington, Seattle, WA, United States,; 2 Department of Global Health, University of Washington, Seattle, WA, United States,; 3 Department of Obstetrics, Gynecology & Reproductive Sciences, Yale University School of Medicine, New Haven, CT, United States

**Keywords:** pregnancy, virus, zika (ZIKV), lymphocytic choriomeningitis virus (LCMV), SARS-CoV-2, COVID-19, anellovirus, placenta

Viral pandemics, like the severe acute respiratory syndrome coronavirus 2 (SARS-CoV-2), are becoming more common and disproportionately impact the health of pregnant individuals and their fetuses. Recently, the probability of an extreme epidemic similar in intensity to the 1918 “Spanish influenza” with roughly 32 million deaths was estimated at 1.9% annually (1). For an individual born in 2000, this estimate translates to a 38% lifetime probability of experiencing an extreme epidemic by 2020. This probability estimate was expected to increase up to three-fold due to human-environment interactions, disease emergence from zoonotic reservoirs, and climate change (1). Depending on the virus, an infection during pregnancy may impart risk for more severe maternal disease, preterm birth, preeclampsia, stillbirth, fetal congenital anomalies and/or a long-term risk for neuropsychiatric disease in the children (2–9). This Research Topic includes scientific manuscripts broadly covering viral infections in pregnancy and their impact on pregnancy outcome and the maternal-placental-fetal immune response. Here, we summarize the articles included in this Research Topic and their importance to the field of translational virology in pregnancy.

Understanding the impact of viral infections in pregnancy on the fetus typically begins with *in vitro* and *in vivo* models with a focus on the placental response to infection. Sheridan et al. reviewed the wide range of current human placental models that include primary trophoblast cell and explant cultures, trophoblast cell lines, and trophoblast stem cells and organoids. This review also discusses how these models may be used to learn about viral trafficking and placental immune defense as well as their strengths and limitations. Since viral infection of the placenta is thought to play a pivotal role in adverse pregnancy outcomes, an important research area is the investigation of placental immunopathology and factors permitting or restricting viral replication at the maternal-fetal interface. Devi Negi et al. explored the role of interleukin-10 (IL-10) in viral clearance from uterine and placental tissues in a murine model of lymphocytic choriomeningitis virus (Armstrong; LCMV-Arm). High IL-10 at the maternal-fetal interface impaired viral clearance suggesting that modulation of IL-10 expression may be beneficial in preventing adverse pregnancy outcomes. In a study of human placentas from women testing positive for COVID-19, Sharps et al. evaluated the distribution of immune cells and found a significant increase in the number of CD163^+^ placental macrophages (Hofbauer cells) and vascularity in the COVID-19 group. The assortment of available *in vitro* and *in vivo* systems for modeling placental viral infections are essential for understanding the role of immune responses in controlling viruses at the maternal-fetal interface.

The Zika virus (ZIKV) pandemic in 2015–2016 was linked to thousands of cases of congenital microcephaly and a wide spectrum of central nervous system anomalies in newborns (10). A series of articles in this Research topic addresses the impact of ZIKV infection on placental cellular processes, immune defenses, and vertical transmission. Guzeloglu-Kayisli et al. reviewed the molecular mechanisms underlying ZIKV infection of placental cells and highlight the role that decidual cells play in the propagation of ZIKV in extravillous cytotrophoblast cells and subsequent transplacental infection. Using a rhesus macaque model and single cell RNA sequencing, Haese et al. found that ZIKV infection induced the stable expression of antiviral genes within CD14^+^ cells of the placenta, which may act to limit ZIKV from propagating in the placenta and causing further tissue injury. McKinney et al. demonstrated that downregulation of autophagy genes and proteins occurs later in ZIKV infection by triangulating data from primary human trophoblast cells, human placentas, and a marmoset model. ZIKV continues to pose a viral threat globally as it is endemic in large geographic regions with sporadic outbreaks and the potential to collapse health care sectors (11). Investigating the molecular and cellular consequences of ZIKV infection during pregnancy and potential pharmaceutical targets to prevent vertical transmission remains a high priority.

A major knowledge gap is the impact of maternal viral infections on placental and fetal development. Many viruses are known to cross the placenta and infect the fetus, such as ZIKV and the ‘TORCH’ infections [varicella-zoster virus, parvovirus B19, rubella virus, cytomegalovirus, herpes simplex virus]. Creisher et al. used an immunocompetent mouse model of ZIKV infection to demonstrate a broad downregulation of transcriptional activity of genes in the placenta that drive tissue morphology, neurological development, cell signaling and inflammation. Maternal infections and immune activation have also been associated with an increased long-term risk in the child for neuropsychiatric disease including autism spectrum disorders (ASD) (3, 12). Sharma and Jash discussed the link between viral infection in pregnancy, uterine immune activation and regulatory T cells in the development of ASD-like fetal brain pathology. This highlights a potential transcriptional mechanism for ZIKV-mediated placental and fetal pathology. Finally, an intriguing article by Goetzl et al. proposed a novel non-invasive method for diagnosing fetal viral infection, and thus viral infection-associated fetal brain injury, through the quantitation of fetal central nervous system extracellular vesicles from maternal serum or plasma. For nearly all viruses with pandemic potential, our knowledge of how infection impacts fetal neurodevelopment is either incomplete or completely unknown.

In addition to highly pathogenic viruses known to adversely impact pregnancy and fetal outcomes, there are also commensal viruses colonizing humans with an unknown impact on pregnancy outcomes. Anelloviruses (family Anelloviridae) are non-enveloped circular single-stranded DNA viruses that are a nearly ubiquitous colonizer of humans and can be found in maternal blood, amniotic fluid, cervical and vaginal fluid, and breast milk. Kyathanahalli et al. presented an intriguing possible link between anellovirus colonization and birth timing through an analysis of what is known regarding the interactions of anelloviruses with other microbes and impact on maternal host defense. The understanding of the vaginal microbiota and innate/adaptive immune crosstalk that contributes to the risk for spontaneous preterm birth continues to evolve (13), which leaves the possibility that immunomodulation by anelloviruses, or other unknown microbes, may change the vaginal immune milieu and increase preterm birth risk.

There is a great need for therapeutic development to mitigate the maternal-placental-fetal injury posed by a viral infection in pregnancy. Viral infections may be associated with an increased risk for preeclampsia, a hypertensive disorder in pregnancy that can be life-threatening for the mother. Indeed, recent data indicates that SARS-CoV-2 infection is strongly associated with the development of preeclampsia (14). Modeling this *in vivo*, preeclamptic-like symptoms have been linked to the activation of the Toll-like receptor 3 (TLR3) pathway by double-stranded RNA (dsRNA), which is produced by positive-strand RNA viruses, like SARS-CoV-2 (15, 16). In this mouse model, Balasubbramanian et al. found that high-dose vardenafil blunted the hypertensive effects of TLR3 activation by the viral dsRNA mimic, Poly(I:C). Drug repurposing combined with new approaches for drug validation in animal models can significantly accelerate the approximate 10–20 year timeline for moving a drug from bench-to-bedside (17). Evaluating potential therapeutics for efficacy in pregnancy is not a challenge embraced by the pharmaceutical industry and one that the reproductive scientific community bears disproportionately (18).

Pandemic preparedness requires an understanding of the impact of a wide array of viruses on maternal-fetal immunity, maternal health, pregnancy outcomes and fetal development. The risk for new viral epidemics and pandemics is increasing with climate change and human-environment interactions. Investigation of the molecular and cellular processes contributing to placental immunopathology and immune defense is the foundation for understanding the effect of viruses on the maternal-fetal dyad, which is essential for the development of clinical approaches to improve maternal and fetal outcomes.
